# Outcome of cancer patients considered for intensive care unit admission in two university hospitals in the Netherlands: the danger of delayed ICU admissions and off-hour triage decisions

**DOI:** 10.1186/s13613-021-00898-2

**Published:** 2021-08-11

**Authors:** Esther N. van der Zee, Dominique D. Benoit, Marinus Hazenbroek, Jan Bakker, Erwin J. O. Kompanje, Nuray Kusadasi, Jelle L. Epker

**Affiliations:** 1 Department of Intensive Care, Erasmus MC University Medical Center, Room Ne-403, Doctor molewaterplein 40, 3015 GD Rotterdam, the Netherlands; 2 Department of Intensive Care, Ghent University Hospital, Ghent, Belgium; 3 Department of Pulmonology and Critical Care, New York University, New York, USA; 4 Department of Pulmonology and Critical Care, Columbia University Medical Center, New York, USA; 5 Department of Intensive Care, Pontificia Universidad Católica de Chile, Santiago, Chile; 6 Department of Intensive Care, University Medical Center Utrecht, Utrecht, the Netherlands

**Keywords:** Intensive Care Unit, Critical care, Triage, Admission decisions, Malignancy, Cancer, Mortality

## Abstract

**Background:**

Very few studies assessed the association between Intensive Care Unit (ICU) triage decisions and mortality. The aim of this study was to assess whether an association could be found between 30-day mortality, and ICU admission consultation conditions and triage decisions.

**Methods:**

We conducted a retrospective cohort study in two large referral university hospitals in the Netherlands. We identified all adult cancer patients for whom ICU admission was requested from 2016 to 2019. Via a multivariable logistic regression analysis, we assessed the association between 30-day mortality, and ICU admission consultation conditions and triage decisions.

**Results:**

Of the 780 cancer patients for whom ICU admission was requested, 332 patients (42.6%) were considered ‘too well to benefit’ from ICU admission, 382 (49%) patients were immediately admitted to the ICU and 66 patients (8.4%) were considered ‘too sick to benefit’ according to the consulting intensivist(s). The 30-day mortality in these subgroups was 30.1%, 36.9% and 81.8%, respectively. In the patient group considered ‘too well to benefit’, 258 patients were never admitted to the ICU and 74 patients (9.5% of the overall study population, 22.3% of the patients ‘too well to benefit’) were admitted to the ICU after a second ICU admission request (delayed ICU admission). Thirty-day mortality in these groups was 25.6% and 45.9%. After adjustment for confounders, ICU consultations during off-hours (OR 1.61, 95% CI 1.09–2.38, p-value 0.02) and delayed ICU admission (OR 1.83, 95% CI 1.00–3.33, p-value 0.048 compared to “ICU admission”) were independently associated with 30-day mortality.

**Conclusion:**

The ICU denial rate in our study was high (51%). Sixty percent of the ICU triage decisions in cancer patients were made during off-hours, and 22.3% of the patients initially considered “too well to benefit” from ICU admission were subsequently admitted to the ICU. Both decisions during off-hours and a delayed ICU admission were associated with an increased risk of death at 30 days. Our study suggests that in cancer patients, ICU triage decisions should be discussed during on-hours, and ICU admission policy should be broadened, with a lower admission threshold for critically ill cancer patients.

**Supplementary Information:**

The online version contains supplementary material available at 10.1186/s13613-021-00898-2.

## Introduction

Overall mortality of cancer patients has decreased over the past decades, due to improvement in early detection and innovative cancer treatments [1, 2]. At the same time, the probability of life-threatening events requiring Intensive Care Unit (ICU) treatment related to these novel therapies has increased [3]. Therefore, intensivists are increasingly confronted with cancer patients for whom ICU admission is requested [4, 5].

Several studies assessed the influence of early versus late ICU admission on outcome in cancer patients [6–12]. However, the majority of these studies focussed on specific subgroups of critically ill cancer patients (e.g., acute respiratory failure or leukemia) [6–9]. To the best of our knowledge, only Thiery et al. compared outcome between cancer patients immediately admitted to the ICU and cancer patients who initially remained in the ward, but were subsequently admitted to the ICU during the same hospitalization [10]. Thus, the effect of triage decisions on outcome in acutely ill cancer patients remains mostly unknown.

In addition, the association between ICU admission consultation conditions, such as ICU consultation during on-hours versus off-hours or the number of physicians involved, and the outcome of patients was not evaluated in these studies. Although it is a common belief that patients admitted to the ICU during off-hours have a higher risk of death [13], this remains a controversial issue [13, 14].

The aim of our study was to evaluate whether ICU admission consultation conditions and ICU triage decisions were associated with 30-day mortality after adjusting for baseline confounders. Furthermore, to explore whether associations found in this analysis persisted over time (at 90 days, 180 days and 1 year).

## Methods

### Participating hospitals

We conducted a retrospective cohort study in two large referral university hospitals in the Netherlands (Erasmus Medical Center, Rotterdam and University Medical Center Utrecht, Utrecht). Both hospitals had a mixed, closed ICU with 56 and 36 beds, respectively. In one of the hospitals, a medium care was present, which could be a suitable alternative for ICU care, for example for administering a low dose of vasopressors. The Netherlands has a healthcare insurance program covering all residents without ICU admission restrictions or restrictions in indicated ICU treatments.

In general, ICU admission was considered when ICU treatment was requested by the referring specialist, either by a junior or senior physician. No ICU triage protocol with predefined ICU admission criteria existed in both hospitals during the study period. The national guideline for ICU triage of critically ill patients in general is outdated (2005) and currently under revision [15]. During our study period, ‘at risk’ patients on the ward were not routinely discussed by oncologists or hematologists and ICU physicians. Referring physicians used the Modified Early Warning Score (MEWS) and clinical assessment for ICU referral decisions. During on-hours, the patient was evaluated by an intensivist, whom subsequently decided whether to admit or reject admission. During off-hours, the patient was evaluated by a fellow (intensivist in training), who subsequently consulted the senior ICU attending. The final decision was made together (fellow and attending). In both hospitals, treatment recommendations for patients denied ICU admission were provided by the intensivist or fellow.

In general, decisions to admit were based on severity of illness and patient-related factors such as age, cancer status, comorbidities and performance status. ICU physicians used these factors to estimate whether an ICU admission would be inappropriate, either because the patient is very likely to recover without intensive care treatment (patients ‘too well to benefit’) or because an ICU admission is very unlikely to prevent death (patients ‘too sick to benefit’).

### Ethics

The institutional review board of the Erasmus Medical Center approved the study. Local approval was obtained for the University Medical Center Utrecht. No additional patient consent was required due to the non-invasive retrospective nature of the study.

### Patient data

Using our Electronic Health Records (EHR), we identified all adult cancer patients for whom ICU treatment was requested from 2016 to 2019. Patients with a planned ICU admission, an emergency surgery or intervention, after cardiopulmonary resuscitation or transferred from another ICU were excluded, as these patients are generally admitted to the ICU without previous discussions between referring physicians and ICU physicians.

Reasons for ICU admission consultation noted in the EHR by the referring specialism were collected (i.e., shock, respiratory insufficiency, altered consciousness, sepsis, acute kidney injury (AKI), high MEWS, hemodynamic instability or other).

Patients who were transferred to another ICU due to bed unavailability were included in the ICU admission group. Patients who were initially denied admission, but admitted after a second request during the same hospitalization were defined as ‘delayed ICU admission’. We collected reasons for ICU denial and acceptance, together with context parameters: (1) time (on-hours or off-hours), (2) place (emergency room, ward or other, such as post-anesthesia care unit), and (3) number of physicians involved in the decision.

### Baseline characteristics

Clinically relevant baseline characteristic such as age, comorbidities and underlying malignancy were collected. A metastatic solid tumor was defined as the presence of cancer cells present in distant organs, determined from the medical charts. We defined controlled cancer as ‘cancer in remission or stable’, while we considered recently diagnosed malignancies and progressive malignancies as “uncontrolled cancer”. We used the Eastern Cooperative Oncology Group Performance Status (ECOG PS) to evaluate performance status 1 month to 14 days before hospital admission and the Charlson Comorbidity Index (CCI) for comorbidities [16]. To evaluate severity of critical illness before the ICU consultation, the MEWS was used, as this score is used in both hospitals by physicians and nurses [17, 18]. Cancer patients were not automatically considered as ‘immunocompromised’. Patients were only considered immunocompromised when they met one of the five National Intensive Care Evaluation criteria: (1) long-term immunosuppressive therapy; (2) corticosteroid use (either short-term high dose or long-term low dose); (3) chemotherapy or radiotherapy in the past year; (4) chemotherapy or radiotherapy for Hodgkin or non-Hodgkin lymphoma at any time before ICU admission; (5) documented humoral or cellular deficiencies.

### Primary and secondary endpoints

The primary objective of this study was to evaluate the association between 30-day mortality, and ICU admission consultation conditions and triage decisions in critically ill cancer patients.

The secondary objectives were the association between 90-day, 180-day and 1-year mortality, and ICU admission consultation conditions and triage decisions.

### Statistical analysis

We categorized the patient population into four groups according to the triage decision: (1) patients considered too well to benefit from ICU treatment, never admitted to the ICU; (2) patients with a delayed ICU admission (initially considered too well to benefit, however, admitted to the ICU after a second ICU request); (3) patients immediately admitted to the ICU and (4) patients considered too sick to benefit from ICU treatment. Descriptive statistics were used to describe patient characteristics. We reported categorical variables as numbers with percentage, and continuous variables as median with 25th–75th interquartile range (IQR). To assess differences between the groups, we used Pearson’s Chi-square tests for categorical variables and the Kruskal–Wallis test for continuous variables. A statistical test with a two-tailed *p* value ≤ 0.05 was considered as significant.

We assessed the association between ICU admission consultation conditions and triage decisions, and the 30-day mortality via logistic regression analysis. We performed a univariable logistic regression analysis, in which we included patient characteristics (e.g., age, gender, cancer type, performance status, etc.), ICU triage decision (i.e., the four groups as described above) and ICU admission consultation characteristics. All variables with a *p* value of < 0.2 in this regression analysis were included in the multivariable model. We tested for an interaction between ICU triage decisions and on-hours consultation, between ICU triage decisions and cancer type, and between metastatic disease and cancer type, all three interaction terms were statistically not significant (*p*-value 0.21, 0.44 and 0.57, respectively).

Finally, we assessed the association between 90-day, 180-day and 1-year mortality, and ICU admission consultation conditions and ICU triage decisions in a similar way. In a post hoc analysis, we assessed the severity of illness, time of ICU consultation (on-hours vs. off-hours) and consult reasons of the second ICU triage decision in patients with delayed ICU admission. Data were analyzed using IBM®SPSS® Statistics 24.0 (IBM, Chicago, IL, USA).

## Results

**Fig. 1:**
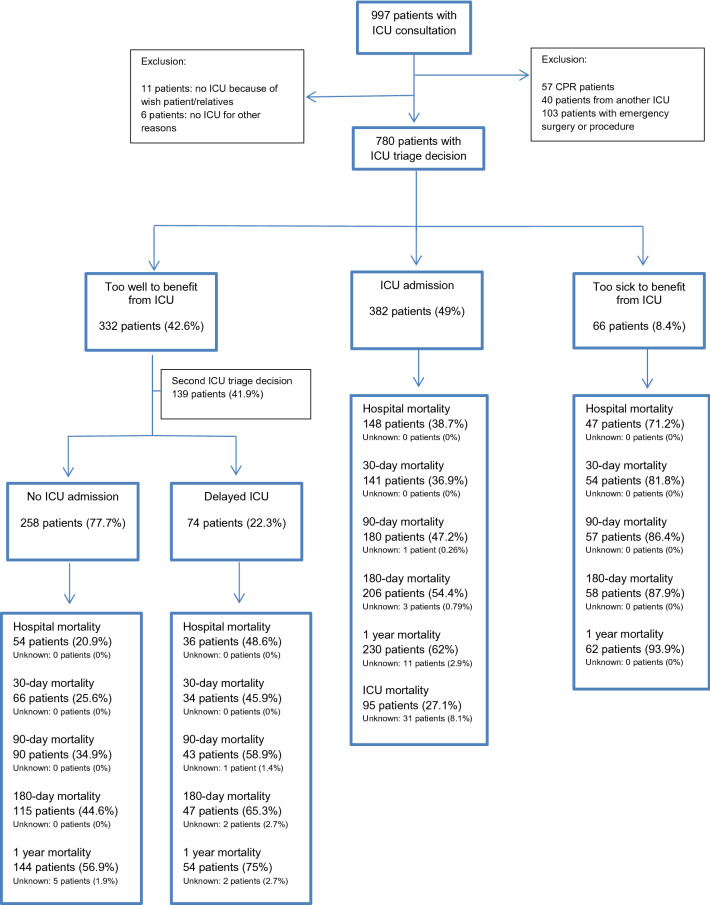
Flowchart study: number patients, ICU triage decisions and outcomes

We included 780 cancer patients with an ICU admission request. Of these patients, 332 (42.6%) were considered ‘too well to benefit from ICU’, 382 (49%) were admitted to the ICU and 66 (8.4%) were considered ‘too sick to benefit from ICU’ (Fig. 1). For 139 patients (41.9%) of the ‘too well to benefit’ group, ICU admission was requested a second time during the same hospitalization. Of the 332 patients initially considered ‘too well to benefit’, 258 patients (77.7%) were never admitted to the ICU, 74 patients (22.3%) were admitted to the ICU after the second request (delayed ICU admission, Fig. 1). The median time between first and second admission request was 1 day [0–2.5].

Patient characteristics, severity of illness scores and admission consultation characteristics of the total study population and the 4 groups are shown in Table 1. The different types of cancer are reported in Additional file 1: Table S1. The ‘too sick to benefit’ group had more patients with solid malignancy, metastatic disease and uncontrolled cancer when compared to the other groups. In addition, the CCI and ECOG performance status were higher in patients ‘too sick to benefit’ than in the other groups. The MEWS was the lowest in patients ‘too well to benefit’, and comparable in the other 3 groups.

**Table 1:** Baseline characteristics by ICU triage decision

	Total study population N = 780	Too well to benefit—No ICU N = 258	Too well to benefit—Delayed ICU N = 74	ICU N = 382	Too sick to benefit N = 66	*p*-value
Age	64 [56–70]	63 [54–69]	66 [59–70]	65 [58–72]	63 [56–70]	0.04*
Male	499 (64%)	166 (64.3%)	44 (59.5%)	248 (64.9%)	41 (62.1%)	0.82
Solid malignancy	503 (64.7%)	163 (63.2%)	41 (55.4%)	244 (63.9%)	55 (83.3%)	0.005*
Hematological malignancy	274 (35.3%)	94 (36.4%)	33 (44.6%)	136 (35.6%)	11 (16.7%)	
Both	3 (0.4%)	1 (0.4%)		2 ( 0.5%)		
Metastatic solid malignancy	245 (31.4%)	75 (29.1%)	20 (27%)	108 (28.3%)	42 (63.6%)	< 0.001*
Cancer recurrence	71 (9.1%)	33 (12.8%)	5 (6.8%)	27 (7.1%)	6 (9.1%)	0.09
Uncontrolled cancer	408 (52.4%)	145 (56.2%)	33 (44.6%)	173 (45.4%)	57 (86.4%)	< 0.001*
CCI	4 [2–6]	4 [2–6]	3 [2–6]	4 [2–6]	6 [4–7]	< 0.001*
ECOG PS before hospital	1 [1–2]	1 [1–2]	1 [1–1]	1 [0–2]	2 [1–3]	< 0.001*
0	181 (23.2%)	54 (20.9%)	17 (23%)	108 (28.3%)	2 (3%)	
1	298 (38.2%)	93 (36%)	39 (52.7%)	148 (38.7%)	18 (27.3%)	
2	191 (24.5%)	74 (28.7%)	15 (20.3%)	81 (21.2%)	21 (31.8%)	
3	90 (11.5%)	31 (12%)	3 (4.1%)	36 (9.4%)	20 (30.3%)	
4	15 (1.9%)	5 (1.9%)	0 (0%)	6 (1.6%)	4 (6.1%)	
Unknown	5 (0.6%)	1 (0.4%)	0 (0%)	3 (0.8%)	1 (1.5%)	
MEWS	5 [3–6]	4 [2–6]	5 [4–7]	5 [4–7]	5 [3–6]	< 0.001*
Unknown	80 (10.3%)	14.(5.4%)	4 (5.4%)	54 (14.1%)	8 (12.1%)	
Cancer treatment before ICU	684 (88%)	223 (86.8%)	65 (87.8%)	343 (90.3%)	53 (80.3%)	0.12
Unknown	3 (0.4%)					
Immunocompromised	560 (71.8%)	199 (77.1%)	49 (66.2%)	265 (69.4%)	47 (71.2%)	0.13
Year ICU consult						0.20
2016	214 (27.4%)	67 (26%)	22 (29.7%)	114 (29.8%)	11 (16.7%)	
2017	204 (26.2%)	62 (24%)	18 (24.3%)	108 (28.3%)	16 (24.2%)	
2018	222 (28.5%)	78 (30.2%)	18 (24.3%)	99 (25.9%)	27 (40.9%)	
2019	140 (17.9%)	51 (19.8%)	16 (21.6%)	61 (16%)	12 (18.2%)	
Earlier ICU admission before consult	77 (9.9%)	24 (9.3%)	8 (10.8%)	45 (11.8%)	0 (0%)	0.03*
One ICU physician	318 (40.8%)	109 (42.2%)	35 (47.3%)	158 (41.4%)	16 (24.2%)	0.007*
Two ICU physicians	385 (49.4%)	130 (50.4%)	37 (50%)	176 (46.1%)	42 (63.6%)	
More than two ICU physicians	77 (9.9%)	19 (7.4%)	2 (2.7%)	48 (12.6%)	8 (12.1%)	
One referring physician (ref)	164 (21.2%)	59 (23%)	19 (26%)	80 (21.2%)	6 (9.1%)	< 0.001*
Two referring physicians	496 (64.2%)	175 (68.1%)	41 (56.2%)	240 (63.7%)	40 (60.6%)	
More than two referring physicians	113 (14.6%)	23 (8.9%)	13 (17.8%)	57 (15.1%)	20 (30.3%)	
Location consult						< 0.001*
Emergency room (ref)	137 (17.6%)	30 (11.6%)	4 (5.4%)	81 (21.2%)	22 (33.3%)	
Ward	483 (61.9%)	197 (76.4%)	55 (74.3%)	190 (49.7%)	41 (62.1%)	
Other	160 (20.5%)	31 (12%)	15 (20.3%)	111 (29.1%)	3 (4.5%)	
On-hours (ref)	315 (40.4%)	95 (36.8%)	27 (36.5%)	163 (42.7%)	30 (45.5%)	0.34
Off-hours	465(59.6%)	163 (63.2%)	47 (63.5%)	219 (57.3%)	36 (54.5%)	
Weekend	244 (31.3%)	77 (29.8%)	25 (33.8%)	118 (30.9%)	24 (36.4%)	0.73
Consult reason						
Shock	150 (19.2%)	30 (11.6%)	5 (6.8%)	103 (27%)	12 (18.2%)	< 0.001*
Respiratory insufficiency	483 (61.9%)	147 (57%)	50 (67.6%)	242 (63.4%)	44 (66.7%)	0.20
Altered consciousness	139 (17.8%)	36 (14.0%)	7 (9.5%)	80 (20.9%)	16 (24.2%)	0.01*
Sepsis	214 (27.4%)	47 (18.2%)	22 (29.7%)	135 (35.3%)	10 (15.2%)	< 0.001*
Acute kidney injury	94 (12.1%)	18 (7%)	10 (13.5%)	60 (15.7%)	6 (9.1%)	0.008*
High MEWS	23 (2.9%)	16 (6.2%)	3 (4.1%)	2 (0.5%)	2 (3%)	0.001*
Hemodynamic instability	280 (35.9%)	86 (33.3%)	21 (28.4%)	152 (39.8%)	21 (31.8%)	0.14
Other	99 (12.7%)	42 (16.3%)	9 (12.2%)	36 (9.4%)	12 (18.2%)	0.05

Table shows data of first ICU consultation of the hospital admission

A *p*-value of < 0.05 is considered significant (marked by an *)

*CCI* Charlson Comorbidity Index, *ECOG PS* Eastern Cooperative Oncology Group Performance Status, *MEWS* Modified Early Warning Score, *On-hours* during dayshift

Patients ‘too sick to benefit’ were more often seen in the emergency room, and more physicians, both referring and ICU physicians, were involved in the decision-making. Reasons for ICU admission consultation differed between groups (Table 1).

In Additional file 2: Table S2, data of patients (e.g., APACHE IV score and length of ICU stay) are shown.

Mortality rates of the 4 groups are shown in Fig. 1. The crude 30-day mortality of patients ‘too well to benefit’ was 30.1%, in patients admitted to the ICU 36.9%, and in patients ‘too sick to benefit’ 81.8% (Fig. 1). The 30-day mortality was 25.6% in patients ‘too well to benefit, never admitted to the ICU’, and 45.9% in patients with a delayed ICU admission.

### Primary and secondary outcome

The results of the univariable and multivariable logistic regression analysis with 30-day mortality as endpoint are provided in Table 2. The following factors of the multivariable analysis were associated with 30-day mortality: age, a hematological type of cancer, uncontrolled cancer, ECOG PS of 2, 3 and 4, MEWS, ICU triage decisions ‘delayed ICU admission’ and ‘too sick to benefit’, ICU consultations during off-hours, ‘altered consciousness’ as reason for ICU admission request and ‘AKI’ as reason for ICU admission request.

**Table 2:** Univariable and multivariable analysis: factors associated with 30-day mortality

	30-day Mortality	Univariable analysis			Multivariable analysis		
		Odds ratio	95% CI	p-value	Odds ratio	95% CI	p-value
Age	–	1.02	1.01–1.03	0.001*	1.04	1.02–1.05	< 0.001*
Male	192 (38.5%)	1.08	0.80–1.46	0.61			
Solid malignancy (ref)	176 (35%)						
Hematological malignancy	117 (42.7%)	1.39	1.02–1.87	0.03*	1.63	1.02–2.60	0.04*
Metastatic disease	101 (41.2%)	1.23	0.91–1.68	0.19	1.05	0.56–1.96	0.89
Cancer recurrence	32 (45.1%)	1.39	0.85–2.27	0.19	1.39	0.74–2.60	0.31
Controlled cancer (ref)	98 (26.4%)						
Uncontrolled cancer	197 (48.3%)	2.60	1.92–3.52	< 0.001*	2.30	1.54–3.41	< 0.001*
CCI	–	1.08	1.01–1.15	0.02*	1.02	0.89–1.15	0.82
ECOG PS before hospital admission							
0 (ref)	47 (26%)						
1	107 (35.9%)	1.60	1.06–2.40	0.02*	1.33	0.81–2.18	0.27
2	82 (42.9%)	2.15	1.38–3.33	0.001*	1.84	1.07–3.19	0.03*
3	44 (48.9%)	2.73	1.61–4.64	< 0.001*	2.40	1.21–4.77	0.01*
4	13 (86.7%)	18.53	4.03–85.19	< 0.001*	12.42	2.34–65.93	0.003*
MEWS		1.13	1.05–1.20	< 0.001*	1.13	1.04–1.23	0.006*
Cancer treatment	252 (36.8%)	0.77	0.50–1.20	0.25			
Immunocompromised	214 (38.2%)	1.09	0.79–1.51	0.61			
ICU triage decision							
ICU admission (ref)	141 (36.9%)						
Too well to benefit—no ICU admission	66 (25.6%)	0.59	0.42–0.83	0.003*	0.66	0.42–1.04	0.08
Too well to benefit—delayed ICU	34 (45.9%)	1.45	0.88—2.40	0.15	1.83	1.00—3.33	0.048*
Too sick to benefit	54 (81.8%)	7.69	3.98–14.87	< 0.001*	7.78	3.38–17.89	< 0.001*
Year ICU consult							
2016 (ref)	78 (36.4%)	–	–	–			
2017	77 (37.7%)	1.06	0.71–1.57	0.78			
2018	87 (39.2%)	1.12	0.76–1.66	0.56			
2019	53 (37.9%)	1.06	0.68–1.65	0.79			
Earlier ICU admission before consult	18 (23.4%)	0.47	0.27–0.81	0.007*	0.82	0.41–1.64	0.57
One ICU physician (ref)	109 (34.3%)						
Two ICU physicians	155 (40.3%)	1.29	0.95–1.76	0.10	0.97	0.64–1.46	0.87
More than two ICU physicians	31 (40.3%)	1.29	0.78–2.15	0.33	0.95	0.48–1.87	0.88
One referring physician (ref)	53 (32.3%)						
Two referring physicians	195 (39.3%)	1.36	0.93–1.97	0.11	1.27	0.80–2.03	0.31
More than two referring physicians	45 (39.8%)	1.39	0.84–2.28	0.20	1.24	0.64–2.38	0.53
Location consult							
Emergency room (ref)	57 (41.6%)	–	–	–			
Ward	188 (38.9%)	0.89	0.61–1.32	0.57	1.42	0.86–2.35	0.18
Other	50 (31.3%)	0.64	0.40–1.03	0.07	1.70	0.90–3.20	0.10
On-hours (ref)	103 (32.7%)						
Off-hours	192 (41.3%)	1.45	1.07–1.95	0.02*	1.61	1.09–2.38	0.02*
Weekend	95 (38.9%)	1.07	0.79–1.46	0.67			
Consult reason							
Shock	68 (45.3%)	2.49	1.48–4.19	0.001*	1.78	0.97–3.26	0.06
Respiratory insufficiency	188 (38.9%)	1.48	1.02–2.17	0.04*	1.18	0.77–1.83	0.45
Altered consciousness	68 (48.9%)	2.12	1.35–3.34	0.001*	1.73	1.06–2.83	0.03*
Sepsis	87 (40.7%)	1.28	0.84–1.97	0.25			
Acute kidney injury	50 (53.2%)	2.70	1.65–4.41	< 0.001*	2.47	1.41–4.34	0.002*
High MEWS	10 (43.5%)	1.93	0.80–4.64	0.14	2.00	0.70–5.72	0.20
Hemodynamic instability	102 (36.4%)	0.69	0.42–1.14	0.15	0.70	0.42–1.18	0.18
Other	34 (35.4%)	1.14	0.70–1.87	0.59			

Table shows data of first ICU triage decision of the hospital admission

A *p*-value of < 0.05 is considered significant (marked by an *)

*CCI* Charlson Comorbidity Index, *ECOG PS* Eastern Cooperative Oncology Group Performance Status, *MEWS* Modified Early Warning Score, *On-hours* during dayshift

We presented in Tables 3 and 4 a comparison between patients immediately admitted to the ICU and patients with a delayed ICU admission, and a comparison between patients with a consultation during on-hours and patients with a consultation during off-hours.

**Table 3:** Differences immediate ICU admission vs. delayed ICU admission

	ICU admission *N* = 382	Too well to benefit—delayed ICU *N* = 74	*p*-value
Age	65 [58–72]	66 [59–70]	0.98
Male	248 (64.9%)	44 (59.5%)	0.37
Solid malignancy	244 (63.9%)	41 (55.4%)	0.15
Hematological malignancy	136 (35.6%)	33 (44.6%)	
Metastatic solid malignancy	108 (28.3%)	20 (27%)	0.83
Cancer recurrence	27 (7.1%)	5 (6.8%)	0.92
Uncontrolled cancer	173 (45.4%)	33 (44.6%)	0.90
CCI	4 [2–6]	3 [2–6]	0.64
ECOG PS before hospital	1 [0–2]	1 [1–1]	0.54
0	108 (28.3%)	17 (23%)	
1	148 (38.7%)	39 (52.7%)	
2	81 (21.2%)	15 (20.3%)	
3	36 (9.4%)	3 (4.1%)	
4	6 (1.6%)	0 (0%)	
Unknown	3 (0.8%)	0 (0%)	
MEWS	5 [4–7]	5 [4–7]	0.13
Unknown	54 (14.1%)	4 (5.4%)	
Cancer treatment before ICU Unknown	343 (90.3%)	65 (87.8%)	0.53
Immunocompromised	265 (69.4%)	49 (66.2%)	0.55
Year ICU consult			0.66
2016	114 (29.8%)	22 (29.7%)	
2017	108 (28.3%)	18 (24.3%)	
2018	99 (25.9%)	18 (24.3%)	
2019	61 (16%)	16 (21.6%)	
Earlier ICU admission before consult	45 (11.8%)	8 (10.8%)	0.82
One ICU physician	158 (41.4%)	35 (47.3%)	0.045*
Two ICU physicians	176 (46.1%)	37 (50%)	
More than two ICU physicians	48 (12.6%)	2 (2.7%)	
One referring physicians	80 (21.2%)	19 (26%)	0.48
Two referring physicians	240 (63.7%)	41 (56.2%)	
More than two referring physicians	57 (15.1%)	13 (17.8%)	
Location consult			< 0.001*
Emergency room	81 (21.2%)	4 (5.4%)	
Ward	190 (49.7%)	55 (74.3%)	
Other	111 (29.1%)	15 (20.3%)	
On-hours	163 (42.7%)	27 (36.5%)	0.32 0.62
Off-hours	219 (57.3%)	47 (63.5%)	
Weekend	118 (30.9%)	25 (33.8%)	
Consult reason			
Shock	103 (27%)	5 (6.8%)	< 0.001*
Respiratory insufficiency	242 (63.4%)	50 (67.6%)	0.49
Altered consciousness	80 (20.9%)	7 (9.5%)	0.02*
Sepsis	135 (35.3%)	22 (29.7%)	0.35
Acute kidney injury	60 (15.7%)	10 (13.5%)	0.63
High MEWS	2 (0.5%)	3 (4.1%)	0.008*
Hemodynamic instability	152(39.8%)	21 (28.4%)	0.06
Other	36 (9.4%)	9 (12.2%)	0.42
Hospital mortality	148 (38.7%)	36 (48.6%)	0.11
30-day mortality	141 (36.9%)	34 (45.9%)	0.14
90-day mortality	180 (47.2%)	43 (58.9%)	0.07
180-day mortality	206 (54.4%)	47 (65.3%)	0.09
1 year mortality	230 (62%)	54 (75%)	0.04*

Table shows data of first ICU consultation of the hospital admission

A *p*-value of < 0.05 is considered significant (marked by an *)

*CCI* Charlson Comorbidity Index, *ECOG PS* Eastern Cooperative Oncology Group Performance Status, *MEWS* Modified Early Warning Score, *On-hours* during dayshift

**Table 4:** Differences on-hours versus off-hours

	On-hours *N* = 315	Off-hours *N* = 465	*p*-value
Age	65 [57–71]	63 [56–70]	0.12
Male	208 (66%)	291 (62.6%)	0.33
Solid malignancy	207 (66.1%)	296 (63.8%)	0.50
Hematological malignancy	106 (33.9%)	168 (36.2%)	
Metastatic solid malignancy	99 (31.4%)	146 (31.4%)	0.99
Cancer recurrence	29 (9.2%)	42 (9.1%)	0.94
Uncontrolled cancer	158 (50.2%)	250 (53.9%)	0.31
CCI	4 [2–6]	4 [2–6]	0.78
ECOG PS before hospital	1 [0–2]	1 [1, 2]	0.07
0	82 (26%)	99 (21.3%)	
1	122 (38.7%)	176 (37.8%)	
2	70 (22.2%)	121 (26%)	
3	37 (11.7%)	53 (11.4%)	
4	3 (1.0%)	12 (2.6%)	
Unknown	1 (0.3%)	4 (0.9%)	
MEWS	5 [3–6]	5 [4–7]	0.99
Unknown	37 (11.7%)	43 (9.2%)	
Cancer treatment before ICU Unknown	284 (90.4%)	400 (86.4%)	0.09
Immunocompromised	231 (73.3%)	329 (71.1%)	0.49
Year ICU consult			0.25
2016	96 (30.5%)	118 (25.4%)	
2017	76 (24.1%)	128 (27.5%)	
2018	93 (29.5%)	129 (27.7%)	
2019	50 (15.9%)	90 (19.4%)	
Earlier ICU admission before consult	35 (11.1%)	42 (9%)	0.34
One ICU physician	184 (58.4%)	134 (28.8%)	< 0.001*
Two ICU physicians	102 (34.2%)	283 (60.9%)	
More than two ICU physicians	29 (9.2%)	48 (10.3%)	
One referring physicians	77 (24.5%)	87 (19%)	0.08
Two referring physicians	187 (59.6%)	309 (67.3%)	
More than two referring physicians	50 (15.9%)	163 (13.7%)	
Location consult			0.32
Emergency room	49 (15.6%)	88 (18.9%)	
Ward	195 (61.9%)	288 (61.9%)	
Other	71 (22.5%)	89 (19.1%)	
Weekend	73 (23.2%)	171 (36.8%)	< 0.001*
Consult reason			
Shock	70 (22.2%)	80 (17.2%)	0.08
Respiratory insufficiency	189 (60%)	294 (63.2%)	0.36
Altered consciousness	61 (19.4%)	78 (16.8%)	0.35
Sepsis	86 (27.3%)	128 (27.5%)	0.95
Acute kidney injury	41 (13%)	53 (11.4%)	0.50
High MEWS	9 (2.9%)	14 (3%)	0.90
Hemodynamic instability	124 (39.4%)	156 (33.5%)	0.10
Other	32 (10.2%)	64 (13.8%)	0.13
Hospital mortality	106 (33.7%)	179 (38.5%)	0.17
30-day mortality	103 (32.7%)	192 (41.3%)	0.02*
90-day mortality	143 (45.4%)	227 (49%)	0.32
180-day mortality	162 (51.6%)	264 (57.3%)	0.12
1 year mortality	188 (61.2%)	302 (66.4%)	0.15

Table shows data of first ICU consultation of the hospital admission

A *p*-value of < 0.05 is considered significant (marked by an *)

*CCI* Charlson Comorbidity Index, *ECOG PS* Eastern Cooperative Oncology Group Performance Status, *MEWS* Modified Early Warning Score, *On-hours* during dayshift

In Additional file 3: Table S3, multivariable analyses of 90-day mortality, 180-day mortality and 1-year mortality are shown. Delayed ICU admission remained associated with mortality, while the effect of on-hours compared to off-hours disappeared.

In Additional file 4: Table S4, MEWS, number of consultations during on-hours and consult reasons of the second ICU admission consultation for patients with a delayed ICU admission are shown. The MEWS of patients during the second consultation was comparable to the MEWS of the first consultation, a second consultation was more often performed during on-hours.

Last, in Additional file 5: Table S5 and Additional file 6: Table S6, crude mortality rates by ICU triage decision for patients with solid cancer and patients with a hematological malignancy separately are shown.

## Discussion

Our study evaluated the association between 30-day mortality, and ICU admission consultation characteristics and triage decisions in cancer patients. After adjustment for confounders, we found a statistically significant relationship between 30-day mortality, and triage decisions and ICU consultation during off-hours. The association between triage decisions and mortality might even persist over time.

In our study, 60% of the ICU triage decisions in cancer patients were made during off-hours, half of the patients for whom ICU admission was requested were immediately admitted to the ICU. Triage decisions were made by two or more ICU and referring physicians in nearly 50% of the patients ‘too well to benefit’ from ICU, and in 80% of the patients ‘too sick to benefit’. Only one patient initially considered ‘too sick to benefit’ was admitted to the ICU after a second ICU triage decision. These results suggest that in our hospitals, patients were often discussed by multiple physicians, before considering them ‘too sick to benefit’ from ICU. However, 22% of the patients initially considered “too well to benefit” from ICU admission (9.5% of the overall population) were subsequently admitted to the ICU after a median of 1 day. Severity of illness, measured by the MEWS, was comparable between the first and second consultation by intensivists in this group, suggesting no overt rapid deterioration in the clinical status of these patients. Worrisome is that both off-hours consultation, and admission to the ICU after initially being considered “too well to benefit” from ICU admission (i.e., delayed ICU admission), were associated with an increased 30-day mortality, even after adjusting for confounders present at the moment of consultation and patient-related confounders. Moreover, the detrimental effect of delayed ICU admission might persist till 1 year after the initial triage decision.

Studies that describe the association between mortality and ICU admission during off-hours in critically ill patients show contradictory results [13, 19, 20]. Where Brunot et al. [13] found that time of admission, especially off-hour admissions, did not influence the prognosis of ICU patients, two other studies [19, 20] found an association between off-hours ICU admissions and hospital mortality. Our results suggest that in cancer patients, the oncologists or hematologists should draw attention of the on-hour intensivist for any situation that could deteriorate rapidly. In addition, although off-hours assessment of patients’ condition seemed not detrimental in the long-term, ICU physicians should be aware of the vulnerability of cancer patients for whom ICU admission during off-hours is requested, and need to consider ICU admission carefully. Daily rounds by a multidisciplinary team are associated with lower mortality among ICU patients [21]. Although not described in studies before, we assume that the outcome of critically ill cancer patients will improve when an ICU physician and a hematologist or oncologist discusses the need of an ICU admission for patients ‘at risk’ during rounds.

In line with previous literature [3, 22–27], age, cancer type (solid or hematological), cancer status, performance status, and severity of critical illness were associated with 30-day mortality in our study and should therefore be taken into consideration during triage decisions. In earlier studies, the association between ICU admission reasons and short-term mortality has been reported [28–30]. Our study adds to these results that altered conscious, AKI and shock as reason for consultation need special consideration, as these are associated with either short-term (Table 2) or long-term mortality (Additional file 3: Table S3).

ICU denial rate in our study was higher than in studies including general patients [31–35], patients with advanced disease [36] or hematological patients [37]. In our study, short-term mortality of patients with immediate ICU admission (i.e., ICU mortality, hospital mortality or 30-day mortality) was variable when compared to other studies reporting similar [29, 38–40] or lower mortality [26, 30, 41]. It would be expected that with a high denial rate, the mortality would be lower. However, when compared to the median-predicted hospital mortality (using the APACHE IV score), the actual hospital mortality was similar (predicted 40.8%, actual hospital mortality 38.7%). The higher mortality when compared to other studies could be explained by differences in case mix (e.g., planned surgical patients were included in those studies as well) and severity of illness.

The 30-day mortality of patients ‘too well to benefit, never admitted to the ICU’ was high (25.6%), especially when compared to the study of Thiery et al. [10] (6% 30-day mortality). However, the study of Thiery et al. [10] had a small sample size and did not show data regarding severity of illness, complicating a direct comparison. In our study, patients were more frequent considered ‘too well to benefit’ (42.6% versus 22.8%), which might explain the difference in mortality. However, mortality of patients with a delayed ICU admission was lower in our study (45.9% versus 61.5%). In addition, approximately 20% of the patients considered ‘too sick to benefit’ was still alive on day 30. Therefore, we assume that decisions to refuse ICU treatment were partly based on ‘long-term’ prognostic factors, where cancer control might be limited, but the patient is not necessarily going to die immediately. Both the high mortality of patients ‘too well to benefit’ from ICU as the lower mortality than expected of patients ‘too sick’ suggest that, despite clinical experience of physicians and current scoring systems, whether or not ICU treatment should be given remains difficult. Errors in judgement of ICU physicians whether ICU admission would be inappropriate care may lead to higher mortality of acutely ill cancer patients. If possible, a MEWS specified to cancer patients should be developed to more adequately address a timely admission and benefit from an ICU admission. In addition, more frequent intra- and interdisciplinary discussions might improve clinical assessment.

The current findings suggest room for a broader admission policy with a lower threshold for critically ill cancer patients in the hospitals that participated in this study. However, bed shortage is associated with ICU refusal [31, 42] and unfortunately, we were not able to collect data on bed availability. We are aware that, unless ICU capacity increases, admitting more cancer patients to the ICU may limit the possibility of ICU admissions for other critically ill patients. A way to deal with these problems is the use of a time-limited ICU trial [43–46]. Previous literature describes that for patients with a solid tumor, an ICU admission of 5 days is sufficient to determine whether a patient will survive the ICU admission [43, 45], and for patients with a hematological malignancy, a maximum of 14 days is sufficient [44]. In order to successfully conduct an ICU trial, clear agreements must be made with oncologists, hematologists, patients and relatives before ICU admission.

### Limitations

First, ICU admission triage varies across hospitals, and in particular high-volume hospitals may have different admission policies. However, we think that our study still shows an important message to many hospitals. Physicians should critically evaluate their own ICU triage policy, and a close collaboration between referring physicians and ICU physicians should be pursued.

Second, where we tried to reduce the heterogeneity by adjusting for patient characteristics such as type of cancer (solid or hematological) and cancer status, this still limits the interpretation of the results.

Third, given the observational design of the study, we cannot rule out residual confounding. We tried to reduce this issue by adjusting for the maximum number of factors which was available at the moment of the consultation. Moreover, limitations with regard to retrospective studies should be acknowledged.

Fourth, we found a significant relationship between ICU triage decisions and 1-year mortality via logistic regression analysis. However, this result might be biased by short-term mortality and residual confounding. Thus, we should interpret this result with caution.

Last, it must be emphasized that by categorizing our cohort according to the triage decisions, we assumed that readmission to the ICU can be predicted with a 100% accuracy by consulting physicians. Although this practice is common in medical research, it does not reflect the real-life situation. Future studies should assess the impact of delayed ICU admission via causal inference techniques, where the longitudinal probability of transitions in care can be taken into account [47].

## Conclusion

The ICU denial rate in our study was high (51%). Sixty percent of the ICU triage decisions in cancer patients were made during off-hours, and 22.3% of the patients initially considered “too well to benefit” from ICU admission were subsequently admitted to the ICU. Both decisions during off-hours and a delayed ICU admission were associated with an increased risk of death at 30 days. Our study suggests that in cancer patients, ICU triage decisions should be discussed during on-hours, and ICU admission policy should be broadened, with a lower admission threshold for critically ill cancer patients.

## Supplementary Information

**Additional file 1.** Supplementary material Table 1; cancer types of patients at ICU admission consultation.

**Additional file 2.** Supplementary material Table 2; ICU data of patients immediately admitted to the ICU.

**Additional file 3.** Supplementary material Table 3; Multivariable analyses; factors associated with 90-day, 180-day and 1 year mortality.

**Additional file 4.** Supplementary material Table 4; characteristics of patients with a delayed ICU triage decision.

**Additional file 5.** Supplementary material Table 5; crude mortality rates of solid cancer patients by ICU triage decision.

**Additional file 6.** Supplementary material Table 6; crude mortality rates of hematological cancer patients by ICU triage decision.

## Data Availability

The datasets used and/or analyzed during the current study are available from the corresponding author on reasonable request.

## Abbreviations

ICU: Intensive Care Unit
OR: Odds ratio
95% CI: 95% Confidence interval
AKI: Acute kidney injury
MEWS: Modified Early Warning Score
EHR: Electronic Health Records
ECOG PS: Eastern Cooperative Oncology Group Performance Status
CCI: Charlson Comorbidity Index
IQR: Interquartile range

## References

[1] Allemani C, Matsuda T, Di Carlo V, Harewood R, Matz M, Niksic M (2018). Global surveillance of trends in cancer survival 2000–14 (CONCORD-3): analysis of individual records for 37 513 025 patients diagnosed with one of 18 cancers from 322 population-based registries in 71 countries. Lancet.

[2] Malvezzi M, Carioli G, Bertuccio P, Rosso T, Boffetta P, Levi F (2016). European cancer mortality predictions for the year 2016 with focus on leukaemias. Ann Oncol.

[3] Azoulay E, Pene F, Darmon M, Lengline E, Benoit D, Soares M (2015). Managing critically Ill hematology patients: time to think differently. Blood Rev.

[4] Azoulay E, Schellongowski P, Darmon M, Bauer PR, Benoit D, Depuydt P (2017). The Intensive Care Medicine research agenda on critically ill oncology and hematology patients. Intensive Care Med.

[5] Shimabukuro-Vornhagen A, Boll B, Kochanek M, Azoulay E, von Bergwelt-Baildon MS (2016). Critical care of patients with cancer. CA Cancer J Clin.

[6] de Montmollin E, Tandjaoui-Lambiotte Y, Legrand M, Lambert J, Mokart D, Kouatchet A (2013). Outcomes in critically ill cancer patients with septic shock of pulmonary origin. Shock.

[7] Lengliné E, Raffoux E, Lemiale V, Darmon M, Canet E, Boissel N (2012). Intensive care unit management of patients with newly diagnosed acute myeloid leukemia with no organ failure. Leuk Lymphoma.

[8] Mokart D, Lambert J, Schnell D, Fouché L, Rabbat A, Kouatchet A (2013). Delayed intensive care unit admission is associated with increased mortality in patients with cancer with acute respiratory failure. Leuk Lymphoma.

[9] Song J-U, Suh GY, Park HY, Lim SY, Han SG, Kang YR (2012). Early intervention on the outcomes in critically ill cancer patients admitted to intensive care units. Intensive Care Med.

[10] Thiery G, Azoulay E, Darmon M, Ciroldi M, De Miranda S, Levy V (2005). Outcome of cancer patients considered for intensive care unit admission: a hospital-wide prospective study. J Clin Oncol.

[11] Hourmant Y, Mailloux A, Valade S, Lemiale V, Azoulay E, Darmon M (2021). Impact of early ICU admission on outcome of critically ill and critically ill cancer patients: a systematic review and meta-analysis. J Crit Care.

[12] Doukhan L, Bisbal M, Chow-Chine L, Sannini A, Brun JP, Cambon S (2017). Respiratory events in ward are associated with later intensive care unit (ICU) admission and hospital mortality in onco-hematology patients not admitted to ICU after a first request. PLoS ONE.

[13] Brunot V, Landreau L, Corne P, Platon L, Besnard N, Buzancais A (2016). Mortality associated with night and weekend admissions to ICU with on-site intensivist coverage: results of a nine-year cohort study (2006–2014). PLoS ONE.

[14] Bhonagiri D, Pilcher DV, Bailey MJ (2011). Increased mortality associated with after-hours and weekend admission to the intensive care unit: a retrospective analysis. Med J Aust.

[15] Nederlandse Vereniging voor Intensive Care-Vitaal bedreigde patiënt 2005. https://www.nvic.nl/richtlijnen/vitaal-bedreigde-patient-2005.

[16] de Groot V, Beckerman H, Lankhorst GJ, Bouter LM (2003). How to measure comorbidity. A critical review of available methods. J Clin Epidemiol.

[17] Burch VC, Tarr G, Morroni C (2008). Modified early warning score predicts the need for hospital admission and inhospital mortality. Emerg Med J.

[18] Jiang J, Yang J, Mei J, Jin Y, Lu Y (2018). Head-to-head comparison of qSOFA and SIRS criteria in predicting the mortality of infected patients in the emergency department: a meta-analysis. Scand J Trauma Resusc Emerg Med.

[19] Kuijsten HA, Brinkman S, Meynaar IA, Spronk PE, van der Spoel JI, Bosman RJ (2010). Hospital mortality is associated with ICU admission time. Intensive Care Med.

[20] Laupland KB, Shahpori R, Kirkpatrick AW, Stelfox HT (2008). Hospital mortality among adults admitted to and discharged from intensive care on weekends and evenings. J Crit Care.

[21] Kim MM, Barnato AE, Angus DC, Fleisher LA, Kahn JM (2010). The effect of multidisciplinary care teams on intensive care unit mortality. Arch Intern Med.

[22] Atramont A, Lindecker-Cournil V, Rudant J, Tajahmady A, Drewniak N, Fouard A, et al. Association of age with short-term and long-term mortality among patients discharged from intensive care units in France. JAMA Netw Open. 2019;2(5):e193215-e. 10.1001/jamanetworkopen.2019.3215 PMC6512465 31074809

[23] Camou F, Didier M, Leguay T, Milpied N, Daste A, Ravaud A (2020). Long-term prognosis of septic shock in cancer patients. Support Care Cancer.

[24] Ehooman F, Biard L, Lemiale V, Contou D, de Prost N, Mokart D (2019). Long-term health-related quality of life of critically ill patients with haematological malignancies: a prospective observational multicenter study. Ann Intensive Care.

[25] Lopez R, Samtani SR, Montes JM, Perez R, Martin MJ, Salazar A (2019). Survival of critically ill oncologic patients requiring invasive ventilatory support: a prospective comparative cohort study with nononcologic patients. J Glob Oncol.

[26] Sauer CM, Dong J, Celi LA, Ramazzotti D (2019). Improved survival of cancer patients admitted to the intensive care unit between 2002 and 2011 at a U.S. Teaching Hospital. Cancer Res Treat.

[27] Vincent F, Soares M, Mokart D, Lemiale V, Bruneel F, Boubaya M (2018). In-hospital and day-120 survival of critically ill solid cancer patients after discharge of the intensive care units: results of a retrospective multicenter study-A Groupe de recherche respiratoire en réanimation en Onco-Hématologie (Grrr-OH) study. Ann Intensive Care.

[28] de Vries VA, Müller MCA, Arbous MS, Biemond BJ, Blijlevens NMA, Kusadasi N (2019). Long-term outcome of patients with a hematologic malignancy and multiple organ failure admitted at the intensive care. Crit Care Med.

[29] Ostermann M, Ferrando-Vivas P, Gore C, Power S, Harrison D (2017). Characteristics and outcome of cancer patients admitted to the ICU in England, Wales, and Northern Ireland and National Trends Between 1997 and 2013. Crit Care Med.

[30] Puxty K, McLoone P, Quasim T, Sloan B, Kinsella J, Morrison DS (2018). Characteristics and outcomes of surgical patients with solid cancers admitted to the intensive care unit. JAMA Surg.

[31] Garrouste-Orgeas M, Montuclard L, Timsit JF, Reignier J, Desmettre T, Karoubi P (2005). Predictors of intensive care unit refusal in French intensive care units: a multiple-center study. Crit Care Med.

[32] Iapichino G, Corbella D, Minelli C, Mills GH, Artigas A, Edbooke DL (2010). Reasons for refusal of admission to intensive care and impact on mortality. Intensive Care Med.

[33] Orsini J, Blaak C, Yeh A, Fonseca X, Helm T, Butala A (2014). Triage of Patients Consulted for ICU Admission During Times of ICU-Bed Shortage. J Clin Med Res.

[34] Sprung CL, Geber D, Eidelman LA, Baras M, Pizov R, Nimrod A (1999). Evaluation of triage decisions for intensive care admission. Crit Care Med.

[35] Vanhecke TE, Gandhi M, McCullough PA, Lazar MH, Ravikrishnan KP, Kadaj P (2008). Outcomes of patients considered for, but not admitted to, the intensive care unit. Crit Care Med.

[36] Escher M, Nendaz M, Scherer F, Cullati S, Perneger T. Physicians' predictions of long-term survival and functional outcomes do not influence the decision to admit patients with advanced disease to intensive care: a prospective study. Palliative Medicine. 2020:269216320963931. 10.1177/0269216320963931 33063607

[37] Azoulay E, Mokart D, Pene F, Lambert J, Kouatchet A, Mayaux J (2013). Outcomes of critically ill patients with hematologic malignancies: prospective multicenter data from France and Belgium—a groupe de recherche respiratoire en reanimation onco-hematologique study. J Clin Oncol.

[38] Bos MM, de Keizer NF, Meynaar IA, Bakhshi-Raiez F, de Jonge E (2012). Outcomes of cancer patients after unplanned admission to general intensive care units. Acta Oncol.

[39] Darmon M, Bourmaud A, Georges Q, Soares M, Jeon K, Oeyen S (2019). Changes in critically ill cancer patients' short-term outcome over the last decades: results of systematic review with meta-analysis on individual data. Intensive Care Med.

[40] Auclin E, Charles-Nelson A, Abbar B, Guérot E, Oudard S, Hauw-Berlemont C (2017). Outcomes in elderly patients admitted to the intensive care unit with solid tumors. Ann Intensive Care.

[41] Zampieri FG, Romano TG, Salluh JIF, Taniguchi LU, Mendes PV, Nassar AP, Jr., et al. Trends in clinical profiles, organ support use and outcomes of patients with cancer requiring unplanned ICU admission: a multicenter cohort study. Intensive Care Med. 2020. 10.1007/s00134-020-06184-2 32770267

[42] Robert R, Reignier J, Tournoux-Facon C, Boulain T, Lesieur O, Gissot V (2012). Refusal of intensive care unit admission due to a full unit: impact on mortality. Am J Respir Crit Care Med.

[43] Lecuyer L, Chevret S, Thiery G, Darmon M, Schlemmer B, Azoulay E (2007). The ICU trial: a new admission policy for cancer patients requiring mechanical ventilation. Crit Care Med.

[44] Monsalvo S, Sevillano B, Innes A, Lasa M, Skinner L, Stumpfle R (2016). The intensive care trial for critically ill onco-haematologic patients: the need for response criteria at 5 days of full treatment to separate good risk patients and avoid futile intensive care interventions. Blood.

[45] Shrime MG, Ferket BS, Scott DJ, Lee J, Barragan-Bradford D, Pollard T (2016). Time-limited trials of intensive care for critically ill patients with cancer: how long is long enough?. JAMA Oncol.

[46] Vink EE, Azoulay E, Caplan A, Kompanje EJO, Bakker J (2018). Time-limited trial of intensive care treatment: an overview of current literature. Intensive Care Med.

[47] Vansteelandt S, Bekaert M, Claeskens G (2012). On model selection and model misspecification in causal inference. Stat Methods Med Res.

