# Supplementary material for: Outcome of cancer patients considered for intensive care unit admission in two university hospitals in the Netherlands: the danger of delayed ICU admissions and off-hour triage decisions
Source: Ann Intensive Care. 2021 Aug 11;11:125. doi: 10.1186/s13613-021-00898-2 (PMC8357904; doi:10.1186/s13613-021-00898-2)
Supplement: Supplementary file 1 — Additional file 1. Supplementary material Table 1; cancer types of patients at ICU admission consultation. [file 13613_2021_898_MOESM1_ESM.docx]

|  | **Total study population**  **N = 780** | **Too well to benefit – No ICU**  **N = 258** | **Too well to benefit- Delayed ICU**  **N = 74** | **ICU**  **N = 382** | **Too sick to benefit**  **N = 66** | **p-value** |
| --- | --- | --- | --- | --- | --- | --- |
| Bladder carcinoma | 16 (2.1%) | 7 (2.7%) | 0 (0%) | 6 (1.6%) | 3 (4.5%) | 0.34 |
| Breast carcinoma | 13 (1.7%) | 5 (1.9%) | 0 (0%) | 6 (1.6%) | 2 (3%) | 0.68 |
| Cholangiocarcinoma | 29 (3.7%) | 4 (1.6%) | 7 (9.5%) | 16 (4.2%) | 2 (3%) | 0.003* |
| Colorectal carcinoma | 78 (10%) | 22 (8.5%) | 8 (10.8%) | 42 (11%) | 6 (9.1%) | 0.49 |
| Esophageal carcinoma | 54 (6.9%) | 17 (6.6%) | 4 (5.4%) | 27 (7.1%) | 6 (9.1%) | 0.99 |
| Gastric carcinoma | 22 (2.8%) | 7 (2.7%) | 1 (1.4%) | 12 (3.1%) | 2 (3%) | 0.90 |
| Hepatocellular carcinoma | 20 (2.6%) | 7 (2.7%) | 1 (1.4%) | 11 (2.9%) | 1 (1.5%) | 0.77 |
| Larynx carcinoma | 12 (1.5%) | 4 (1.6%) | 0 (0%) | 7 (1.8%) | 1 (1.5%) | 0.73 |
| Lung carcinoma | 62 (7.9%) | 26 (10%) | 2 (2.7%) | 24 (6.3%) | 10 (15.2%) | 0.06 |
| Malignancy of central nervous system | 16 (2.1%) | 5 (1.9%) | 0 (0%) | 7 (1.8%) | 4 (6.1%) | 0.22 |
| Melanoma | 12 (1.5%) | 0 (0%) | 2 (2.7%) | 7 (1.8%) | 3 (4.5%) | 0.06 |
| Mesothelioma | 3 (0.4%) | 1 (0.4%) | 1 (1.4%) | 1 (0.3%) | 0 (0%) | 0.42 |
| Neuroendocrine tumor | 20 (2.6%) | 10 (3.9%) | 1 (1.4%) | 8 (2.1%) | 1 (1.5%) | 0.36 |
| Ovarian cancer | 17 (2.2%) | 8 (3.1%) | 2 (2.7%) | 6 (1.6%) | 1 (1.5%) | 0.48 |
| Pancreatic carcinoma | 30 (3.8%) | 7 (2.7%) | 6 (8.1%) | 16 (4.2%) | 1 (1.5%) | 0.04* |
| Prostate carcinoma | 17 (2.2%) | 5 (1.9%) | 1 (1.4%) | 11 (2.9%) | 0 (0%) | 0.39 |
| Renal carcinoma | 10 (1.3%) | 3 (1.2%) | 2 (2.7%) | 4 (1%) | 1 (1.5%) | 0.58 |
| Sarcoma | 16 (2.1%) | 3 (1.2%) | 0 (0%) | 11 (2.9%) | 2 (3%) | 0.30 |
| Testicular cancer | 9 (1.2%) | 6 (2.3%) | 2 (2.7%) | 1 (0.3%) | 0 (0%) | 0.03* |
| Other solid tumour | 52 (6.7%) | 17 (6.6%) | 3 (4.1%) | 28 (7.3%) | 4 (6.1%) | 0.74 |
| Unknown solid tumour | 13 (1.7%) | 4 (1.6%) | 0 (0%) | 4 (1%) | 5 (7.6%) | 0.01* |
|  |  |  |  |  |  |  |
| Acute lymphocytic leukemia | 14 (1.8%) | 4 (1.6%) | 1 (1.4%) | 9 (2.4%) | 0 (0%) | 0.66 |
| Acute myeloid leukemia | 82 (10.5%) | 33 (12.8%) | 11 (14.9%) | 35 (9.2%) | 3 (4.5%) | 0.46 |
| Chronic lymphocytic leukemia | 11 (1.4%) | 5 (1.9%) | 0 (0%) | 6 (1.6%) | 0 (0%) | 0.52 |
| Chronic myeloid leukemia | 11(1.4%) | 2 (0.8%) | 2 (2.7%) | 7 (1.8%) | 0 (0%) | 0.54 |
| Hodgkin Lymphoma | 3 (0.4%) | 1 (0.4%) | 0 (0%) | 2 (0.4%) | 0 (0%) | 0.88 |
| Multiple Myeloma | 38 (4.9%) | 6 (2.3%) | 2 (2.7%) | 27 (4.9%) | 3 (4.5%) | 0.008* |
| Myelodysplastic syndrome | 21 (2.7%) | 10 (3.9%) | 2 (2.7%) | 6 (2.6%) | 3 (4.5%) | 0.02* |
| Non-Hodgkin lymphoma | 75 (9.6%) | 30 (11.6%) | 10 (13.5%) | 34 (9.4%) | 1 (1.5%) | 0.35 |
| Other hematological malignancy | 26 (3.3%) | 6 (2.3%) | 5 (6.8%) | 15 (3%) | 0 (0%) | 0.27 |
| Unknown hematological malignancy | 2 (0.3%) | 0 (0%) | 0 (0%) | 1 (0.4%) | 1 (1.5%) | 0.008* |

**Supplementary material table 1; Malignancy types**

- Table shows data of first ICU triage decision of the hospital admission
- A p-value of < 0.05 is considered significant (marked by an *)
- Some patients have more than one malignancy at the same time
