# Supplementary material for: Outcome of cancer patients considered for intensive care unit admission in two university hospitals in the Netherlands: the danger of delayed ICU admissions and off-hour triage decisions
Source: Ann Intensive Care. 2021 Aug 11;11:125. doi: 10.1186/s13613-021-00898-2 (PMC8357904; doi:10.1186/s13613-021-00898-2)
Supplement: Supplementary file 2 — Additional file 2. Supplementary material Table 2; ICU data of patients immediately admitted to the ICU. [file 13613_2021_898_MOESM2_ESM.docx]

|  | **ICU patients**  **N = 382** |
| --- | --- |
| SOFA score^a^  Unknown | 8 [5-11]  118 (30.9%) |
| APACHE IV score^b^  Unknown | 84 [63-103]  116 (30.4%) |
| APACHE IV^b^ predicted mortality  Unknown | 40.8% [20.3%-69.7%]  116 (30.4%) |
| Mechanical ventilation | 213 (60.7%) |
| Renal replacement therapy | 55 (15.7%) |
| Vasopressors | 238 (67.8%) |
| CPR^c^ during ICU | 13 (3.7%) |
| Length of ICU stay (days) | 3 [1-7] |
| Written TLD^d^ | 81 (23.1%) |
| Initiation comfort care^e^ | 100 (28.5%) |

**Supplementary material Table 2; ICU data of patients immediately admitted to the ICU**

- Table shows data of first ICU consultation of the hospital admission
- Data from 31 patients are missing, they were transferred to another ICU.

1. SOFA Score: Sequential Organ Failure Assessment Score
2. APACHE IV score: Acute Physiology and Chronic Health Evaluation IV score
3. CPR: cardiopulmonary resuscitation
4. TLD: treatment limitation decisions
5. Comfort care: the withdrawal of life-sustaining ICU treatment combined with the initiation of palliative medications when indicated.
