# Supplementary material for: Outcome of cancer patients considered for intensive care unit admission in two university hospitals in the Netherlands: the danger of delayed ICU admissions and off-hour triage decisions
Source: Ann Intensive Care. 2021 Aug 11;11:125. doi: 10.1186/s13613-021-00898-2 (PMC8357904; doi:10.1186/s13613-021-00898-2)
Supplement: Supplementary file 3 — Additional file 3. Supplementary material Table 3; Multivariable analyses; factors associated with 90-day, 180-day and 1 year mortality. [file 13613_2021_898_MOESM3_ESM.docx]

|  | **Multivariable analysis** | **90-day**  **mortality** |  | **Multivariable analysis** | **180-day**  **mortality** |  | **Multivariable analysis** | **1 year**  **mortality** |  |
| --- | --- | --- | --- | --- | --- | --- | --- | --- | --- |
|  | Odds ratio | 95% CI | p-value | Odds ratio | 95% CI | p-value | Odds ratio | 95% CI | p-value |
| Age | 1.03 | 1.01-1.04 | 0.001* | 1.02 | 1.01-1.03 | 0.009* | 1.02 | 1.01-1.04 | 0.005* |
| Solid malignancy (ref)  Hematological malignancy | 1.42 | 0.90-2.24 | 0.13 | 1.28 | 0.82-2.00 | 0.27 | 1.04 | 0.65-1.66 | 0.86 |
| Metastatic disease | 0.87 | 0.47-1.61 | 0.66 | 1.18 | 0.64-2.18 | 0.59 | 0.74 | 0.38-1.43 | 0.37 |
| Cancer recurrence | 1.48 | 0.80-2.75 | 0.21 | 1.15 | 0.63-2.10 | 0.66 | 1.33 | 0.69-2.58 | 0.40 |
| Controlled cancer (ref)  Uncontrolled cancer | 2.88 | 1.93-4.30 | <0.001* | 2.59 | 1.74-3.84 | <0.001* | 3.26 | 2.13-4.98 | <0.001* |
| CCI^a^ | 1.06 | 0.93-1.20 | 0.38 | 1.03 | 0.91-1.17 | 0.66 | 1.07 | 0.93-1.22 | 0.33 |
| ECOG PS^b^ before hospital admission  0 (ref)  1  2  3  4 | 1.29  2.09  3.02  8.32 | 0.81-2.03  1.25-3.52  1.56-5.86  1.62-42.76 | 0.28  0.005*  0.001*  0.01* | 0.91  2.17  2.41  4.98 | 0.59-1.42  1.31-3.61  1.25-4.64  0.97-25.52 | 0.69  0.003*  0.009*  0.05 | 0.81  2.02  1.86  5.59 | 0.52-1.27  1.19-3.45  0.93-3.71  0.78-56.11 | 0.35  0.01*  0.08  0.08 |
| MEWS^c^ | 1.10 | 1.01-1.20 | 0.03* | 1.11 | 1.02-1.20 | 0.02* | 1.10 | 1.01-1.20 | 0.03* |
| Cancer treatment | 1.11 | 0.62-1.99 | 0.72 | 1.03 | 0.58-1.85 | 0.92 | 1.24 | 0.67-2.31 | 0.50 |
| ICU triage decision  ICU admission (ref)  Too well to benefit-no ICU admission  Too well to benefit- delayed ICU  Too sick to benefit | 0.56  1.96  4.21 | 0.37-0.87  1.08- 3.57  1.77-10.00 | 0.009*  0.03*  0.001* | 0.68  1.92  3.59 | 0.45-1.04  1.05- 3.51  1.45-8.90 | 0.07  0.04*  0.006* | 0.87  2.39  4.80 | 0.56-1.34  1.25-4.60  1.57-14.72 | 0.52  0.009*  0.006* |
| Year ICU consult  2016 (ref)  2017  2018  2019 | 1.02  1.27  1.11 | 0.63-1.66  0.79-2.04  0.65-1.90 | 0.92  0.33  0.72 | 1.17  1.22  1.11 | 0.73-1.88  0.76-1.94  0.65-1.89 | 0.51  0.41  0.71 | 1.04  1.25  1.25 | 0.64-1.70  0.77-2.03  0.69-2.21 | 0.87  0.37  0.48 |
| Earlier ICU admission before consult | 0.68 | 0.36-1.29 | 0.23 | 0.82 | 0.45-1.49 | 0.52 | 0.86 | 0.47-1.54 | 0.60 |
| One ICU physician (ref)  Two ICU physicians  More than two ICU physicians | 1.03  1.30 | 0.69-1.52  0.69-2.47 | 0.89  0.42 | 1.02  1.76 | 0.70-1.50  0.92-3.36 | 0.92  0.09 | 1.13  1.43 | 0.76-1.69  0.73-2.78 | 0.55  0.30 |
| Location consult  Emergency room (ref)  Ward  Other | 1.36  1.25 | 0.84-2.20  0.68-2.29 | 0.22  0.47 | 1.25  1.08 | 0.77-2.02  0.59-1.96 | 0.45  0.80 | 0.94  1.00 | 0.56-1.58  0.53-1.88 | 0.81  0.99 |
| On-hours^d^ (ref)  Off-hours | 1.10 | 0.76-1.60 | 0.61 | 1.14 | 0.79-1.64 | 0.48 | 1.14 | 0.78-1.67 | 0.50 |
| Consult reason  Shock  Respiratory insufficiency  Altered consciousness  Acute kidney injury  High MEWS^c^  Hemodynamic instability | 2.18  1.21  1.82  1.72  1.34  0.63 | 1.22-3.89  0.80-1.83  1.11-2.98  0.99-3.00  0.47-3.86  0.39-1.03 | 0.008*  0.37  0.02*  0.06  0.59  0.06 | 2.37  1.36  1.32  1.95  2.44  0.63 | 1.33-4.22  0.90-2.05  0.81-2.16  1.11-3.44  0.87-6.83  0.39-1.01 | 0.003*  0.14  0.26  0.02*  0.09  0.06 | 2.56  1.28  1.49  1.50  2.25  0.55 | 1.40-4.67  0.83-1.96  0.87-2.53  0.83-2.69  0.76-6.63  0.33-0.89 | 0.002*  0.26  0.14  0.18  0.14  0.02* |

**Supplementary material Table 3; Multivariable analyses; factors associated with 90-day, 180-day and 1 year mortality.**

- Table shows data of first ICU consultation of the hospital admission
- A p-value of < 0.05 is considered significant (marked by an *)

1. CCI: Charlson comorbidity index
2. ECOG PS: Eastern Cooperative Oncology Group Performance Status
3. MEWS: Modified Early Warning Score
4. On-hours: during dayshift
