# Supplementary material for: Outcome of cancer patients considered for intensive care unit admission in two university hospitals in the Netherlands: the danger of delayed ICU admissions and off-hour triage decisions
Source: Ann Intensive Care. 2021 Aug 11;11:125. doi: 10.1186/s13613-021-00898-2 (PMC8357904; doi:10.1186/s13613-021-00898-2)
Supplement: Supplementary file 4 — Additional file 4. Supplementary material Table 4; characteristics of patients with a delayed ICU triage decision. [file 13613_2021_898_MOESM4_ESM.docx]

| **Too well to benefit- Delayed ICU**  **N = 74** | **First triage decision** | **Second triage decision** |
| --- | --- | --- |
| MEWS^a^  Unknown | 5 [4-7]  4 (5.4%) | 5 [4-7]  7 (9.5%) |
| On-hours^b^  Off-hours first | 27 (36.5%)  47 (63.5%) | 41 (55.4%)  33 (44.6%) |
| Consult reason  Shock  Respiratory insufficiency  Altered consciousness  Sepsis  Acute kidney injury  High MEWS^a^  Hemodynamic instability  Other | 5 (6.8%)  50 (67.6%)  7 (9.5%)  22 (29.7%)  10 (13.5%)  3 (4.1%)  21 (28.4%)  9 (12.2%) | 15 (20.3%)  50 (67.6%)  11 (14.9%)  29 (39.2%)  11 (14.9%)  0 (0%)  26 (35.1%)  9 (12.2%) |

**Supplementary material Table 4; characteristics delayed ICU triage decision**

1. MEWS: Modified Early Warning Score
2. On-hours: during dayshift
