# Supplementary material for: Outcome of cancer patients considered for intensive care unit admission in two university hospitals in the Netherlands: the danger of delayed ICU admissions and off-hour triage decisions
Source: Ann Intensive Care. 2021 Aug 11;11:125. doi: 10.1186/s13613-021-00898-2 (PMC8357904; doi:10.1186/s13613-021-00898-2)
Supplement: Supplementary file 5 — Additional file 5. Supplementary material Table 5; crude mortality rates of solid cancer patients by ICU triage decision. [file 13613_2021_898_MOESM5_ESM.docx]

|  | **Total population of solid cancer patients**  **N = 503** | **Too well to benefit – No ICU**  **N = 163** | **Too well to benefit- Delayed ICU**  **N = 41** | **ICU**  **N = 244** | **Too sick to benefit**  **N = 55** | **p-value** |
| --- | --- | --- | --- | --- | --- | --- |
| Hospital mortality  Missing | 167 (33.2%)  0 (0%) | 23 (14.1%)  0 (0%) | 19 (46.3%)  0 (0%) | 86 (35.2%)  0 (0%) | 39 (70.9%)  0 (0%) | <0.001* |
| 30-day mortality  Missing | 176 (35%)  0 (0%) | 30 (18.4%)  0 (0%) | 17 (41.5%)  0 (0%) | 84 (34.4%)  0 (0%) | 45 (81.8%)  0 (0%) | <0.001* |
| 90-day mortality  Missing | 224 (44.5%)  2 (0.4%) | 47 (28.8%)  0 (0%) | 21 (52.5%)  1 (2.4%) | 108 (44.4%)  1 (0.4%) | 48 (87.3%)  0 (0%) | <0.001* |
| 180-day mortality  Missing | 266 (52.9%)  5 (1%) | 67 (41.1%)  0 (0%) | 23 (59%)  2 (4.9%) | 127 (52.7%)  3 (1.2%) | 49 (89.1%)  0 (0%) | <0.001* |
| 1 year mortality  Missing | 309 (61.4%)  12 (2.4%) | 85 (52.8%)  2 (1.2%) | 29 (74.4%)  2 (4.9%) | 144 (61%)  8 (3.3%) | 51 (92.7%)  0 (0%) | <0.001* |

**Supplementary material Table 5; crude mortality rates of solid cancer patients by ICU triage decision**

- Table shows data of first ICU consultation of the hospital admission
- A p-value of < 0.05 is considered significant (marked by an *)
- ICU mortality of ICU patients: 57 (25.1%). Missing: 17 (7%)
- 3 patients with both solid and hematological cancer were excluded from analysis
