# Supplementary material for: Outcome of cancer patients considered for intensive care unit admission in two university hospitals in the Netherlands: the danger of delayed ICU admissions and off-hour triage decisions
Source: Ann Intensive Care. 2021 Aug 11;11:125. doi: 10.1186/s13613-021-00898-2 (PMC8357904; doi:10.1186/s13613-021-00898-2)
Supplement: Supplementary file 6 — Additional file 6. Supplementary material Table 6; crude mortality rates of hematological cancer patients by ICU triage decision. [file 13613_2021_898_MOESM6_ESM.docx]

|  | **Total population of hematological cancer patients**  **N = 274** | **Too well to benefit – No ICU**  **N = 94** | **Too well to benefit- Delayed ICU**  **N = 33** | **ICU**  **N = 136** | **Too sick to benefit**  **N = 11** | **p-value** |
| --- | --- | --- | --- | --- | --- | --- |
| Hospital mortality  Missing | 117 (42.7%)  0 (0%) | 31 (33%)  0 (0%) | 17 (51.5%)  0 (0%) | 61 (44.9%)  0 (0%) | 8 (72.7%)  0 (0%) | 0.03* |
| 30-day mortality  Missing | 117 (42.7%)  0 (0%) | 35 (37.2%)  0 (0%) | 17 (51.5%)  0 (0%) | 56 (41.2%)  0 (0%) | 9 (81.8%)  0 (0%) | 0.03* |
| 90-day mortality  Missing | 143 (52.2%)  0 (0%) | 42 (44.7%)  0 (0%) | 22 (66.7%)  0 (0%) | 70 (51.5%)  0 (0%) | 9 (81.8%)  0 (0%) | 0.03* |
| 180-day mortality  Missing | 157 (57.3%)  0 (0%) | 47 (50%)  0 (0%) | 24 (72.7%)  0 (0%) | 77 (56.6%)  0 (0%) | 9 (81.8%)  0 (0%) | 0.046* |
| 1 year mortality  Missing | 178 (65%)  6 (2.2%) | 58 (63.7%)  3 (1.1%) | 25 (75.8%)  2 (4.9%) | 84 (63.2%)  3 (2.2%) | 11 (100%)  0 (0%) | 0.05 |

**Supplementary material Table 6; crude mortality rates of hematological cancer patients by ICU triage decision**

- Table shows data of first ICU consultation of the hospital admission
- A p-value of < 0.05 is considered significant (marked by an *)
- ICU mortality of ICU patients: 37 (30.3%). Missing: 14 (10.3%)
- 3 patients with both solid and hematological cancer were excluded from analysis
